# Hemodynamic effect of supra-annular implantation of SAPIEN 3 balloon expandable valve

**DOI:** 10.1007/s12928-024-01040-2

**Published:** 2024-09-06

**Authors:** Masakazu Yasuda, Kazuki Mizutani, Kyohei Onishi, Naoko Onishi, Kosuke Fujita, Masafumi Ueno, Atsushi Okamura, Yoshitaka Iwanaga, Genichi Sakaguchi, Gaku Nakazawa

**Affiliations:** 1 Division of Cardiology, Sakurabashi Watanabe Advanced Healthcare Hospital, OSAKA, Japan; 2 Department of Cardiology, Sapporo Cardio vascular Clinic, 8-1, Kita 49 jyo, Higashi 16 jyo, Higashi-ku, Sapporo, 007-0849 Japan; 3 Division of Cardiology, Department of Internal Medicine, Kindai University Faculty of Medicine, OSAKA, Japan; 4 Department of Cardiovascular Surgery, Kindai University Faculty of Medicine, OSAKA, Japan

**Keywords:** Transcatheter aortic valve replacement, Hemodynamic performance, Balloon expandable valve, Supra-annular implantation

## Abstract

The hemodynamic impact of the implantation depth for balloon-expandable valves is under-investigated, especially with higher implantation techniques. We assessed the hemodynamic performance of supra-annular SAPIEN 3 valve implantation. This retrospective study involved consecutive patients who underwent transcatheter aortic valve replacement (TAVR) using the SAPIEN 3. The device implantation depth and transcatheter heart valve (THV) leaflet-nadir position were angiographically analyzed, and supra-annular implantation was defined as a higher leaflet-nadir position than the original annular line. The Doppler hemodynamic status was evaluated at patient discharge. Among 184 patients, 120 (65%) underwent supra-annular implantation, and their mean implantation depth was significantly lower than that of intra-annular implantation (1.1 vs. 5.2 mm, *p* < 0.001). No patients developed valve embolization or coronary occlusion, and none required the TAV-in-TAV procedure. Two (1.6%) patients in the supra-annular implantation group had a mild or greater paravalvular leak. Echocardiography demonstrated that supra-annular implantation had better hemodynamic performance, showing a larger indexed effective orifice area (iEOA) compared with intra-annular implantation (1.09 vs. 0.97 cm^2^/m^2^, *p* < 0.01). There was a weak but negative correlation between the implantation depth and iEOA (*r* =  − 0.27, *p* < 0.01). Moderate or severe prosthesis–patient mismatch (PPM) was found in 35.9% of the intra-annular group and 9.2% of supra-annular of the supra-annular group (*p* < 0.01). In the multivariable analysis, supra-annular implantation was an independent predictor of better THV function (iEOA > 0.85). Supra-annular SAPIEN 3 implantation provides beneficial hemodynamic effects and reduces the PPM risk.

**Graphical abstract:**

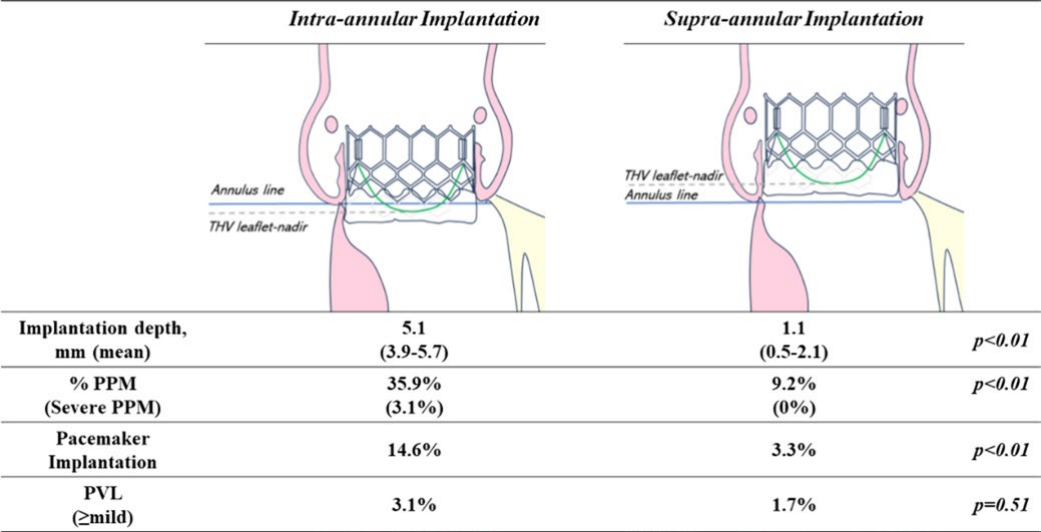

**Supplementary Information:**

The online version contains supplementary material available at 10.1007/s12928-024-01040-2.

## Introduction

Transcatheter aortic valve replacement (TAVR) is a well-established treatment for older adults with severe aortic stenosis, and its indications are expanding to include patients with low surgical risk [1, 2]. Technological developments of the devices, such as smaller device profiles and the evolution of the outer skirt, as well as technical refinements of the procedure have reduced the periprocedural complication rate and improved in-hospital and long-term outcomes [3–5].

Among several transcatheter heart valves (THVs), the SAPIEN series of intra-annular balloon-expandable (BE) valves (Edwards Lifesciences, Irvine, CA, USA) and the EVOLUT series of supra-annular self-expandable valves (Medtronic, Minneapolis, MN, USA) have been used most widely since publication of the excellent results obtained in several randomized controlled trials. Supra-annular THVs reportedly provide better hemodynamic performance with a lower transvalvular pressure gradient, larger effective orifice area (EOA), and lower incidence of patient–prosthesis mismatch (PPM) compared with intra-annular BE THVs [6–8]. Whichever type of valve is used, however, ensuring the optimal THV implantation depth is an important factor to obtain excellent hemodynamic outcomes and avoid pacemaker implantation after TAVR [9, 10]. Deep THV implantation increases the risk of permanent pacemaker implantation (PPI) and more severe paravalvular leak (PVL). Several studies have demonstrated that a higher implantation technique using newer-generation devices is associated with high efficacy and safety and a low incidence of PPI [11, 12]. By contrast, excessively high valve implantation may increase the difficulty of coronary access and TAV-in-TAV procedures.

A previous study investigating the implantation depth for a BE THV demonstrated that the implant position was deeper in patients with severe PPM than in those with no PPM (4.0 vs. 3.5 mm, respectively; *p* = 0.028), suggesting that the implantation depth is associated with the hemodynamic performance of THVs [13]. By contrast, a study of a newer-generation BE THV used in a higher implantation technique with a mean depth of 1.5 mm did not show beneficial effects on hemodynamic performance compared with the conventional technique with a depth of 3.5 mm [12]. However, few reports have focused on the relationship between the implantation depth and hemodynamic performance, especially when using BE THVs. Moreover, the effects and safety of much higher THV deployment techniques are unclear.

We, therefore, investigated the feasibility and hemodynamic performance of BE-TAVR using a supra-annular position.

## Material and methods

### Study population

From May 2017 to December 2021, a total of 186 consecutive patients with severe aortic stenosis undergoing TAVR at Kindai University Hospital (Osakasayama, Japan) using the SAPIEN 3 BE valve were enrolled in this study. Of these, we excluded one patient who complicated annulus rupture followed by surgical conversion and retrieved THV, and one patent died because of ascending aorta dissection with rupture. A total of 184 patents were included for prospective analysis. The study was conducted in accordance with the 1964 Declaration of Helsinki and its later amendments and was approved by the Ethics Committee of Kindai University Faculty of Medicine, Osakasayama, Japan.

### Angiographic analysis

**Fig. 1:**
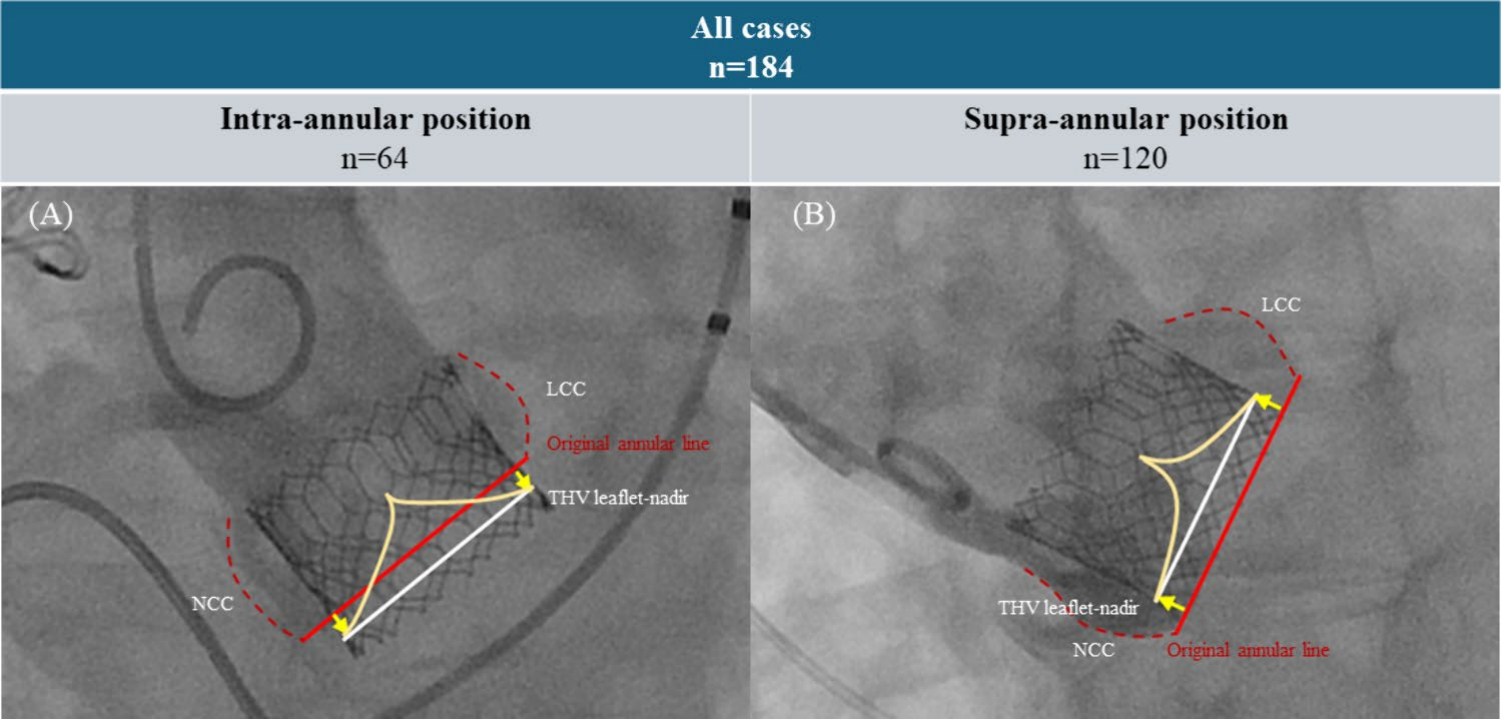
Angiographic assessment. The transcatheter heart valve (THV) leaflet-nadir position, device implantation depth, and THV diameter were angiographically calculated. **A** Intra-annular implantation was defined as a lower THV leaflet-nadir position than the original annular line. **B** Supra-annular implantation was defined as a lower leaflet-nadir position than the original annular line

The device implantation depth from the annular plane, the THV leaflet-nadir depth (i.e., the distance from the nadir of the prosthesis leaflets to the original annular plane), and the THV diameter were angiographically analyzed using post-implantation coplanar aortography with Kada-View software (Photron M&E Solutions Inc., Tokyo, Japan). The implantation depth was measured as the distance from the lower end of the THV stent frame to the bottom of the non-coronary cusp (NCC) and left coronary cusp. The THV leaflet-nadir position was angiographically determined and mostly confirmed to be at half a stent cell above the bottom edge. The THV leaflet-nadir depth was calculated as the nadir of the prosthesis leaflets to the original annular plane. Supra-annular implantation was defined as a higher THV leaflet-nadir position than the original annular plane, and intra-annular implantation was lower than the original annular plane. The THV diameter was calculated as the length of the stent frame at the leaflet-nadir position (Fig. 1).

### Implantation procedures: high implantation technique

**Fig. 2:**
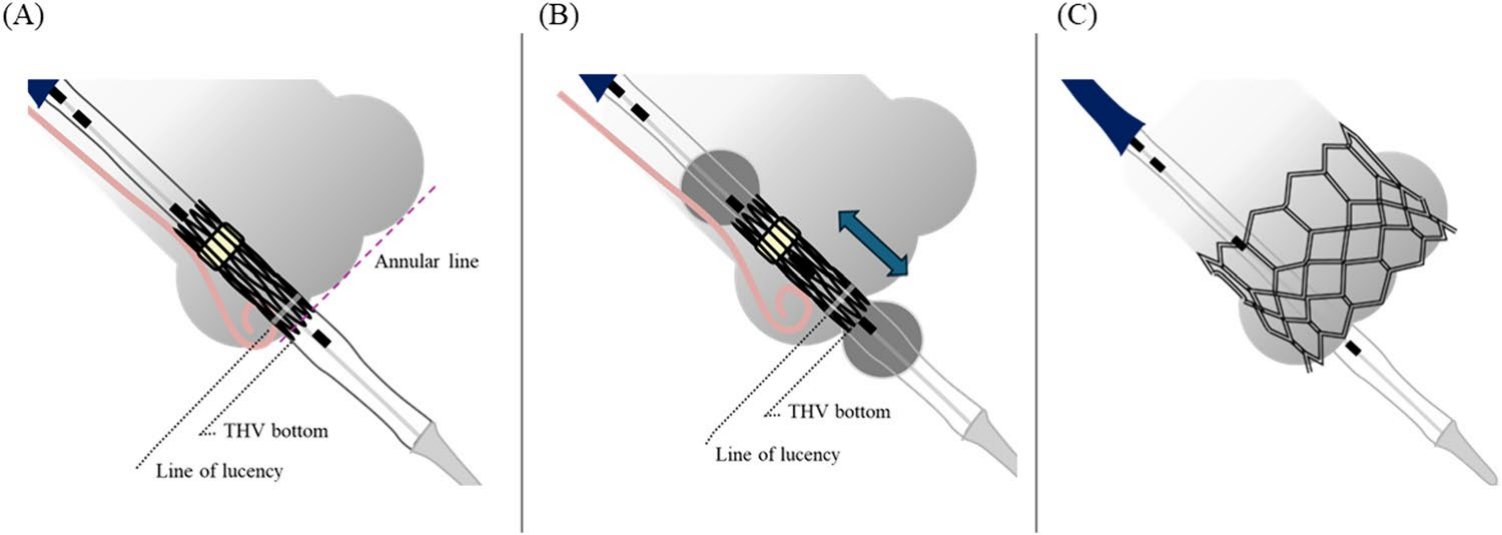
High implantation technique. **A** The bottom edge of the transcatheter heart valve (THV) was placed at the bottom of the non-coronary cusp (NCC) in the pre-implantation phase. **B** After slight inflation, the THV edge was fine-tuned to match the bottom of the NCC bottom. **C** Slow balloon inflation was performed within 20 s, targeting the NCC zero position

To avoid conduction disturbances, we used the original high implantation technique targeting 0 mm of the THV depth deployment. Briefly, before the implantation phase, the bottom edge of the prosthesis was positioned at the bottom of the NCC using a pig-tail catheter placed at the NCC nadir in the coplanar view. Notably, the pre-implantation THV position is higher than that determined using the line of lucency technique [14]. After slight inflation showing a “dog-bone” silhouette, the THV position was fine-tuned and the THV was slowly deployed within 20 s (Fig. 2 and Video). This implantation technique was employed from May 2020 to avoid conduction disturbance.

### Hemodynamic evaluation and outcome

Echocardiography was performed at baseline before the TAVR procedure and at patient discharge, and the results were analyzed in accordance with the American Society of Echocardiography standards for echocardiography guidelines [15]. PVL was measured by Doppler echography using a unified five-grade scheme. Quantitative evaluation of the THV function included flow velocity and pressure gradients and the indexed EOA (iEOA). The Doppler velocity index (DVI) was calculated as the ratio of the velocity–time integral in the left ventricular outflow tract and in the aortic valve. The iEOA and DVI were mainly used to analyze THV hemodynamic performance and PPM. The iEOA was classified into three grades as follows: favorable (> 0.85 cm^2^/m^2^), intermediate (0.65–0.85 cm^2^/m^2^), and poor (< 0.65 cm^2^/m^2^). Normal function of the post-implantation aortic prosthesis is indicated by a DVI of > 0.35 [16], and a previous report showed that a DVI of > 0.50 is associated with a favorable long-term outcome [17]. In accordance with current guidelines and previous reports [15, 17], we applied the following DVI classification: favorable (> 0.50), intermediate (0.50–0.35), and poor (< 0.35). Clinical outcomes, mortality, and device success were evaluated based on the Valve Academic Research Consortium 3 criteria [18].

### Statistical analysis

The study population was divided into intra-annular and supra-annular BE-TAVR groups. Continuous variables are presented as median and interquartile range, and categorical variables are presented as numbers and percentages. Differences in continuous and categorical variables among the groups were compared using the Wilcoxon rank-sum test and the chi-square test, respectively. Pearson’s χ^2^ test or Fisher’s exact test was used to assess differences in categorical variables. Correlations between continuous variables were tested using the Pearson correlation coefficient. Univariate or multivariate logistic regression analyses were performed to identify the risk factors for a favorable DVI, iEOA, and valve performance. A *P* value of < 0.05 was considered statistically significant. Statistical analyses were performed using JMP v.13.0 (SAS Institute, Cary, NC, USA).

## Results

### Study population and procedural characteristics

Among all 184 patients, the median age was 85 (81–88) years, 62.2% of patients were women, and the median Society of Thoracic Surgeons risk score was 6.1%. A total of 120 patients underwent THV implantation in the supra-annular position. The patients’ baseline characteristics are listed in Table 1. Compared with the intra-annular group, the supra-annular group had a higher proportion of men (45.2% vs. 23.8%, *p* < 0.05) and a higher body surface area (1.47 vs. 1.35 m^2^, *p* < 0.05). There were no significant differences in age, Society of Thoracic Surgeons score, or serum B-type natriuretic peptide level between the two groups. Aortic valve calcium volume was significantly higher in supra-annular group. Baseline electrocardiogram showed a higher rate of pre-existing right bundle branch block in intra-annular group.

**Table 1:** Baseline characteristics

	Overall (*n* = 184)	Intra-annular (*n* = 64)	Supra-annular (*n* = 120)	*p* value
Age, years	85 (81–88)	86 (82–90)	84 (81–88)	0.09
Male, *n* (%)	70 (38.0)	15 (23.8)	54 (45.2)	0.007
BSA, m^2^	1.44 (1.33–1.56)	1.35 (1.29–1.50)	1.47 (1.36–1.59)	0.01
LVEF, %	65 (56–70)	64 (55–69)	65 (57–70)	0.57
AVA, cm^2^	0.72 (0.59–0.84)	0.70 (0.56–0.82)	0.75 (0.61–0.86)	0.18
BNP, pg/dl	228.1 (106.3–502.0)	262.7 (118.9–557.6)	206.9 (98.7–489.3)	0.88
STS score, %	6.0 (4.1–9.4)	6.4 (4.3–8.4)	5.8 (3.8–10.1)	0.61
Hemodialysis, *n* (%)	18 (9.7)	2 (3.1)	16 (13.2)	0.02
Urgent or emergent, *n* (%)	37 (20.1)	12 (18.5)	26 (21.4)	0.66
Annulus area on CT, cm^2^	406.4 (369.2–474.3)	392.4 (353.4–449.7)	416.0 (378.9–475.6)	0.09
Oversizing ratio, %	7.9 (3.3–13.6)	9.81 (2.8–14.7)	7.5 (3.4–13.1)	0.41
Aortic valve calcium score, mm^3^	766 (488–1197)	621 (407–1015)	804 (522–1283)	0.01
*Electrocardiographic characteristics*				
Left bundle branch block, *n* (%)	6 (3.2)	2 (3.1)	4 (3.3)	0.93
Right bundle branch block, *n* (%)	27 (14.6)	14 (21.8)	13 (10.8)	0.04
Prior pacemaker implantation, *n* (%)	10 (5.4)	6 (9.3)	4 (3.3)	0.09

Data presented as n (%) or interquartile range (25%–75%)

*BSA* body surface area, *LVEF* left ventricular ejection fraction, *AVA* aortic valve area, *BNP* brain natriuretic peptide, *STS* score, Society of Thoracic Surgery score, *CT* computed tomography

Echocardiographic parameters were also similar between the two groups. Overall, 92.3% of patients underwent a transfemoral approach, 3.8% underwent a subclavian approach, and 1.6% underwent a transapical approach. In both the intra-annular and supra-annular groups, a 23-mm prosthesis was most often implanted, followed by a 26-mm prosthesis. Although a 26-mm prosthesis was used in 41.0% of patients in the supra-annular group, it was used in only 24.5% in the intra-annular group. There was variation in the implanted device sizes between the two groups. On the baseline computed tomography (CT) image, the mean annulus area tended to be larger in the supra-annular group (416.0 vs. 392.4 mm^2^). To assess the device size selection, the oversizing ratio was calculated as the implanted TVH size/annulus area on the pre-procedural CT image, and no significant difference was found between the two groups (Table 2). Pre-dilation was more often performed in the intra-annular group (15.1% vs. 58.7%, *p* < 0.05), whereas post-dilatation was more frequently performed in the supra-annular group (90.7% vs. 41.2%, *p* < 0.05). Regarding the inflation volume, 70% of overall patent had underfilling at THV deployment, and 85% of patent with post-dilatation was underfilling at THV deployment. In patent with post-dilatation, 79% of patent had increased inflation volume at post-dilatation, however, 71% were still underfilling at final inflation volume. Median inflation volume of THV deployment and final inflation were  -2 ml and -1 ml in a supra-annular group, respectively, that was significantly lower compared with a supra-annular group.

**Table 2:** Procedural characteristics and angiographic assessment of transcatheter heart valve

	Overall (*n* = 184)	Intra-annular (*n* = 64)	Supra-annular (*n* = 120)	*p* value
Access site, *n* (%) Femoral Apical Subclavian	171 (92.9) 3 (1.6) 10 (5.4)	59 (92.1) 2 (3.1) 2 (4.6)	112 (93.3) 1 (0.8) 7 (5.8)	0.50
Valve Size, *n* (%) 20 mm 23 mm 26 mm 29 mm	9 (4.8) 99 (53.8) 66 (35.8) 10 (5.4)	7 (10.9) 35 (54.6) 16 (25.0) 6 (9.3)	2 (1.6) 64 (53.3) 50 (41.6) 4 (3.3)	< 0.01
Pre-dilatation, *n* (%)	55 (29.8)	37 (57.8)	18 (15.0)	< 0.01
Post-dilatation, *n* (%)	136 (73.9)	27 (42.1)	109 (90.8)	< 0.01
Inflation volume, nominal ± ml THV deploy PBD final	− 2 (− 2–0) − 1 (− 2–0) − 1 (− 1.5–0)	0 (− 2–0) − 1 (− 2–0) 0 (− 1.8–0)	− 2 (− 2.3–1) − 1 (− 2–0) − 1 (− 1.5–0)	< 0.01
				0.63
				0.01
Leaflet-nadir position, mm	− 1.55 (0.75–2.89)	1.29 (0.62–2.23)	− 2.57 (− 3.32–1.60)	< 0.01
Implantation depth NCC, mm LCC, mm Mean, mm	1.66 (0.64–3.92) 2.45 (0.97–4.70) 2.24 (0.85–4.08)	4.57 (3.51–5.85) 5.26 (4.37–6.81) 5.11 (3.95–5.77)	0.94 (0.28–1.66) 1.40 (0.55–2.42) 1.14 (0.51–2.13)	< 0.01
				< 0.01
				< 0.01
THV-annulus angle,	2.17 (0.35–4.78)	1.84 (0.24–4.62)	2.49 (0.41–4.93)	0.38
Risk plane height, mm Risk plane above STJ, *n* (%)	15.7 (13.8–17.5) 86 (48.5)	13.0 (11.6–15.1) 12 (19.6)	16.5 (10.7–15.1) 74 (63.7)	< 0.01
				< 0.01

Data presented as n (%) or interquartile range (25%–75%)

*NCC* non-coronary cusp, *LCC* left-coronary cusp, *THV* transcatheter heart valve, *STJ* Sinotubular junction

### Procedural result and hemodynamic outcome

The median leaflet nadir position was 2.5 mm above the native annular line in the supra-annular group, whereas it was 1.2 mm below the line in the intra-annular group. The median depth in the supra-annular group was 1.1 mm, and that in the intra-annular group was 5.1 mm.

**Fig. 3:**
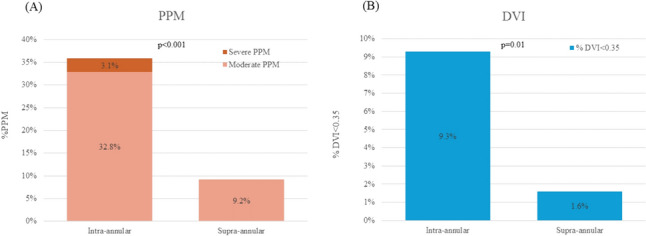
Comparison of intra-annular and supra-annular implantation in the incidence of moderate or severe prosthesis–patient mismatch (PPM). **A** PPM was observed in 35.9% in the intra-annular group and 9.2% in supra-annular group (*p* < 0.01). **B** DVI of < 0.35 was confirmed in 9.3% of patients in the intra-annular group and two 1.6% of patients in the supra-annular group

No patients developed THV embolization, required the TAV-in-TAV procedure, or developed acute coronary occlusion in this study. The PPI ratio was significantly lower in the supra-annular implantation group (3.3% vs. 14.6%, *p* < 0.01). No patients had moderate or severe PVL. Two patients had mild or greater PVL in the supra-annular group, and this proportion of patients was not significantly different from that in the intra-annular group (1.6% vs. 3.1%, *p* = 0.51). There were no differences in 30-day mortality and stroke. On postoperative Doppler echocardiography, the peak aortic jet velocity and mean pressure gradient showed no significant difference between the two groups. However, the median iEOA was 1.09 (0.96–1.27) cm^2^/m^2^ in the supra-annular group and 0.97 (0.81–1.18) cm^2^/m^2^ in the intra-annular group (*p* < 0.05). Additionally, the DVI was significantly higher in the supra-annular group than in the intra-annular group (0.51 vs. 0.47, *p* < 0.05) (Table 3). Two (3.1%) patients had a severe PPM (iEOA of < 0.65 cm^2^/m^2^) in the intra-annular group compared with no patients in the supra-annular group (Fig. 3). Moderate PPM (iEOA of 0.65–0.85 cm^2^/m^2^) was observed in 21 patents (32.8%) in the intra-annular group and 11 patents (9.2%) in the supra-annular group (*p* < 0.01). A DVI of < 0.35 was confirmed in six (9.3%) patients in the intra-annular group and two (1.6%) patients in the supra-annular group.

**Table 3:** Procedural and echocardiographic outcomes

	Intra-annular (*n* = 64)	Supra-annular (*n* = 120)	*p* value
*Procedural outcome*			
Stroke, *n* (%)	0 (0)	1 (0.8)	0.46
Major vascular complication, *n* (%)	2 (3.1)	4 (3.3)	0.94
Coronary occlusion, *n* (%)	0 (0)	0 (0)	–
PPI, *n*, (%)	9 (14.6)	4 (3.3)	< 0.01
Failure to Intended valve performance *n*, (%)	5 (7.8)	3 (2.5)	0.10
30 days mortality, *n* (%)	1 (1.5)	3 (2.5)	0.67
*Echocardiographic outcome*			
LVEF, %	65 (59–70)	65 (58–70)	0.99
PVL > mild, *n* (%)	2 (3.1)	2 (1.7)	0.51
V peak, m/s	2.3 (2.1–2.7)	2.3 (2.1–2.5)	0.14
Mean transvalvular PG, mmHg	11.0 (9.0–14.7)	11.0 (9.0–13.0)	0.26
EOA, cm^2^	1.40 (1.12–1.64)	1.64 (1.39–1.91)	< 0.01
LVOT VTI,	21.7 (16.0–25.0)	21.9 (18.1–25.9)	0.10
SV, ml	60.0 (50.0–75.0)	69.8 (58.4–80.1)	< 0.01
Indexed EOA mean, cm^2^/m^2^	0.97 (0.81–1.18)	1.09 (0.96–1.27)	< 0.01
DVI, mean	0.47 (0.40–0.57)	0.51 (0.45–0.61)	0.014

Data presented as n (%) or interquartile range (25%–75%)

*PPI* permanent pacemaker implantation, *LVEF* left ventricular ejection fraction, *PVL* paravalvular leak, *PG* pressure gradients, *EOA* effective orifice area, *DVI* doppler velocity index

In the device size-based analysis, the EOA of supra-annular implantation was significantly higher in 23-mm, 26-mm, and 29-mm valves compared with intra-annular implantation (Table 4). The mean THV diameter in the supra-annular group was larger than that in the intra-annular group for each THV size (20 mm: 19.9 vs. 20.2 mm, p = 0.74; 23 mm: 22.4 vs. 22.9 mm, *p* < 0.01; 26 mm: 25.2 vs. 25.9 mm, *p* = 0.01; and 29 mm: 27.6 vs. 29.6 mm, *p* = 0.02).

**Table 4:** Doppler hemodynamics for different transcatheter aortic valve prostheses

	Sapien3 20 mm			Sapien3 23 mm			Sapien3 26 mm			Sapien3 29 mm		
Valve position n	Intra- (7)	Supra- (2)	*p* value	Intra- (35)	Supra- (64)	*p* value	Intra- (15)	Supra- (50)	*p* value	Intra- (6)	Supra- (4)	*p* value
Peak velocity, m/s	2.8 (2.3–3.0)	2.9 (2.9–3.0)	0.35	2.5 (2.1–2.7)	2.4 (2.1–2.7)	0.89	2.3 (2.0–2.6)	2.2 (2.0–2.3)	0.12	2.1 (1.8–2.4)	2.2 (1.6–2.3)	0.86
Mean systolic gradient, mmHg	13 (10–17)	18 (18–18)	0.19	13 (9–15)	12 (9–15)	0.97	10 (8–13)	10 (8–12)	0.16	9.5 (7–12)	10 (6–11)	0.66
EOA, cm^2^	1.19 (1.04–1.31)	1.32 (1.31–1.33)	0.54	1.32 (1.10–1.61)	1.49 (1.34–1.64)	< 0.01	1.62 (1.42–1.82)	1.81 (1.68–2.02)	0.01	1.49 (1.40–1.71)	1.90 (1.68–2.44)	0.04
THV diameter, mm	19.9 (19.0–20.9)	20.2 (19.4–20.9)	0.74	22.6 (21.9–23.1)	23.5 (23.0–24.0)	< 0.01	25.5 (23.7–26.5)	25.9 (25.3–26.5)	0.01	27.8 (26.6–28.8)	29.4 (28.9–30.5)	0.02

Data presented as interquartile range (25%–75%)

Abbreviations: EOA, effective orifice area; THV, transcatheter heart valve

**Fig. 4:**
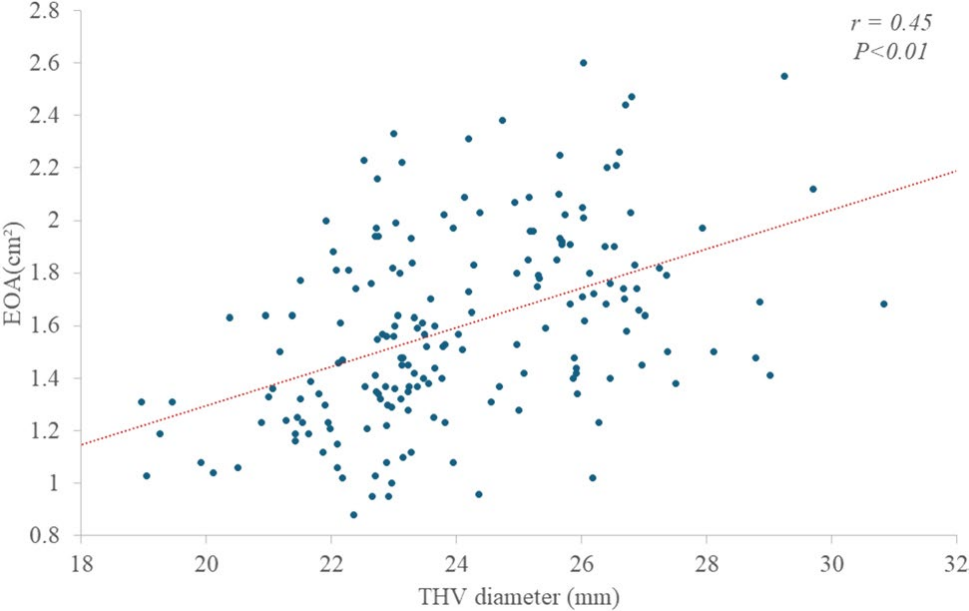
Correlation between transcatheter heart valve (THV) diameter and effective orifice area (EOA). THV diameter was correlated with the EOA (*r* = 0.45, *p* < 0.01)

**Fig. 5:**
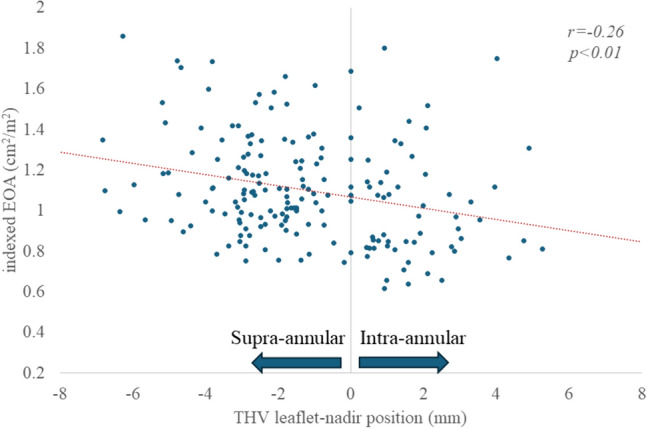
Correlation between transcatheter heart valve nadir-position and indexed effective orifice area (iEOA). The leaflet-nadir position was weakly correlated with the indexed EOA (*r* =  − 0.26, *p* < 0.01)

**Fig. 6:**
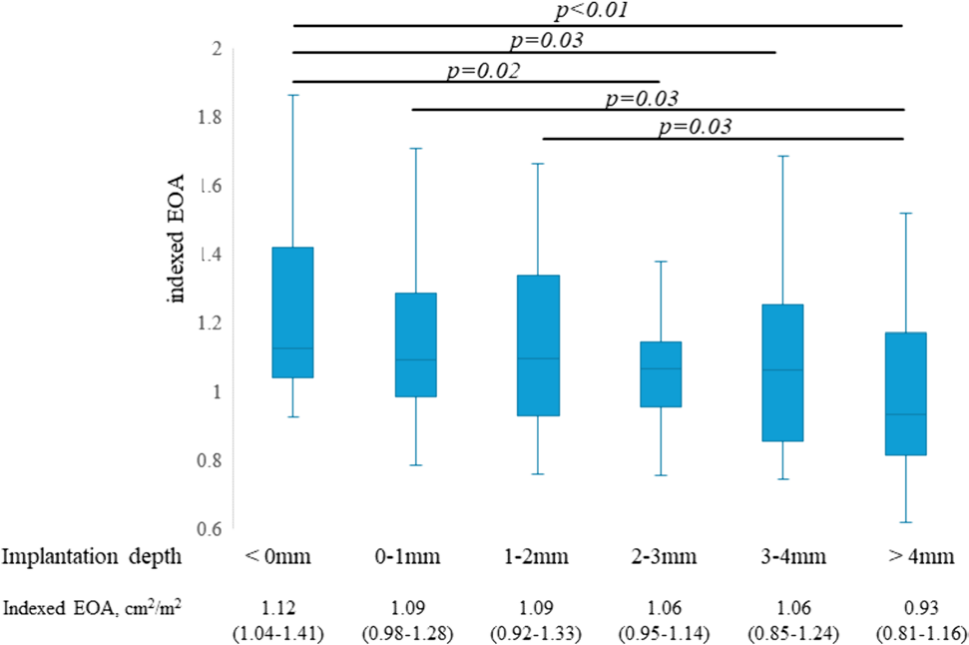
Hemodynamic performance by transcatheter heart valve implantation depth. The indexed effective orifice area (iEOA) was significantly higher in the < 0-mm depth group than those in the 2–3 mm, 3–4 mm, and > 4-mm depth groups. The worst iEOA was confirmed in the > 4-mm depth group, that was significantly lower than that in the < 0-mm, 0–1 mm, and 1–2 mm depth groups

The THV diameter was correlated with EOA (r = 0.45, *p* < 0.01) (Fig. 4). A weak negative correlation was found between the leaflet-nadir position and the DVI (*r* = 0.22, *p* < 0.01) and iEOA (*r* =  − 0.26, *p* < 0.01) (Fig. 5). In the analysis with classification of the implantation depth, a THV depth of < 0 mm (deployed above native annulus) had a superior iEOA and DVI compared with THV depths of 2.0–3.0 mm and > 3.0 mm (Fig. 6).

The results of the univariate analysis for predictors of an iEOA of > 0.85 as indicative of favorable valve function are shown in Table 5. In the multivariate analysis, supra-annular implantation was an independent predictor of an iEOA of > 0.85 (odds ratio, 6.19; 95% confidence interval [CI], 2.32–17.40; *p* < 0.01). In the other model including inflation volume, supra-annular implantation was an independent predictor (supramental Table 2). Similar results were obtained when a DVI of > 0.50 was applied for favorable valve function (odds ratio, 5.0; 95% CI, 1.01–37.5; *p* < 0.05).

**Table 5:** Predictor for favorable transcatheter heart valve function (indexed EOA > 0.85)

	Univariate analysis		Multivariate analysis	
	OR (95% CI)	*p*-value	OR (95% CI)	*p*-value
Age	0.99 (0.92–1.06)	0.76	1.00 (0.92–1.09)	0.75
Male	0.62 (0.26–1.36)	0.24	0.47 (0.15–1.30)	0.15
BSA (per 0.1m^2^ decrease)	1.13 (0.92–1.37)	0.21	1.37 (1.06–1.82)	0.01
Oversizing ratio	1.02 (0.97–1.07)	0.35	1.01 (0.96–1.07)	0.57
Reduced EF (< 50%)	0.67 (0.25–1.99)	0.45	0.65 (0.20–2.31)	0.49
Pre-dilatation	0.39 (0.18–0.85)	0.02	0.41 (0.12–1.29)	0.12
Post-dilatation	1.72 (0.76–3.79)	0.19	0.42 (0.18–1.36)	0.11
THV supra-annular implantation	5.55 (2.54–12.82)	< 0.01	6.19 (2.32–17.4)	< 0.01
23 mm valve (vs 20 mm valve)	0.53 (0.13–2.71)	0.42	–	–
26 mm valve (vs 20 mm valve)	0.23 (0.04–1.31)	0.09	–	–
26 mm valve (vs 23 mm valve)	0.44 (0.16–1.06)	0.06	–	–
29 mm valve (vs 20 mm valve)	0.85 (0.11–6.25)	0.87	–	–
29 mm valve (vs 23 mm valve)	1.59 (0.32–6.28)	0.53	–	–
29 mm valve (vs 26 mm valve)	3.61 (0.66–16.71)	0.12	–	–

*BSA* body surface area, *EF* ejection fraction *THV* transcatheter heart valve

## Discussion

The three main findings of this study are as follows. First, the iEOA and DVI after TAVR were significantly higher in the supra-annular BE-TAVR group. Second, THV diameter was significantly lager in supra-annular BE-TAVR. Third, supra-annular BE-TAVR is an independent predictor of favorable THV function (iEOA of > 0.85).

Few reports have focused on the implantation depth and valve function of TAVR, particularly in BE valves such as the SAPIEN 3. In contrast to our data, a recent report by Sammour et al. [12] showed no hemodynamic advantage when performing high implantation of the SAPIEN 3 valve. By contrast, a retrospective analysis of the implanted device position in 969 patients with the SAPIEN 3 valve showed that no severe PPM occurred in the high implantation position, whereas the rate was 13.8% in the correct position and 21.9% in the low position (*p* = 0.049) [13]. Our depth-control implantation technique also lowers the implantation depth, showing that 85% of patients had the SAPIEN 3 valve implanted in the supra-annular position. The mean THV depth in the supra-annular implantation group was 1.1 mm, which represents much higher implantation than in a previous report (1.5–2.6 mm) [11, 13, 19]. In our population, the distribution of THV deployment depth was as follows: 21 (11.3%) patients had the THV deployed above the annular line, 33 (17.8%) at 0.0–1.0 mm, 34 (18.9%) at 1.0–2.0 mm, 26 (14.0%) at 2.0–3.0 mm, 22 (11.9%) at 3.0–4.0 mm, and 48 (26.0%) at > 4.0 mm. Among these, the THV depth above the annular line had the best hemodynamic performance with respect to the iEOA, and the > 4-mm depth had the worst hemodynamic result. Our data indicate that the THV position is associated with hemodynamic performance, and implantation above the annular line may provide the best hemodynamic performance. It is reported that supra-annular design THV could gain more EOA than intra-annular design THV [6–8]. Similar to this mechanism, we believe that greater EOA can be obtained in the SAPIEN series with an intra-annular design by having the valve function in the supra-annular position.

We found that the THV frame expansion was greater in the supra-annular group for the 23-, 26-, and 29-mm valves. In a recent experimental study of the valve-in-valve technique, Simonato et al. [20] evaluated valve function according to the THV implantation depth. The implantation depth was positively correlated with the mean gradient and negatively correlated with the EOA. Notably, supra-annular implantation had a low mean gradient of 3.7% with better leaflet motion and a better EOA. Although valve-in-valve procedures involve different pathologies and conditions, it is reasonable to consider that a higher THV position has less impact on THV under-expansion and leaflet immobility.

In this study, most patients in the supra-annular group underwent balloon post-dilatation (BPD), which is reportedly associated with greater THV expansion and better hemodynamics [21, 22]. On sub-analysis for BPD, 20 mm valves are used less frequently and 29 mm valve are used more frequently in patent with BPD (supplemental Table 1). There was no significant difference in baseline BSA, annulus area and oversizing ratio. However, our sub-analysis showed no association between the iEOA or DVI with or without BPD (iEOA: 1.07 with BPD vs. 1.04 without BPD, *p* = 0.14; DVI: 0.51 with BPD vs. 0.50 without BPD, *p* = 0.88, respectively). Beside PBD, inflation volume may also have a post-operative hemodynamic performance. Inflation volume at THV deployment was significantly lower in supra-annular group compared with intra-annular group. Additionally, the final inflation volume was significantly lower in supra-annular grope. Nevertheless, THV expansion, iEOA and DVI were significantly higher in supra-annular group. Comparison of inflation volume showed no significant differences between the underfill, nominal and overfill groups for iEOA.

By contrast, supra-annular implantation had significantly better hemodynamic performance than intra-annular implantation (DVI: 0.51 vs. 0.47, *p* < 0.05). Only the supra-annular position was an independent factor for favorable valve function (iEOA of > 0.85 cm^2^/m^2^ and DVI of > 0.5) in the multivariate analyses including BPD and inflation volume. These results suggest that supra-annular implantation is a more important factor for better THV hemodynamic function than is BPD.

Several studies using an iEOA of < 0.65 cm^2^/m^2^ to indicate severe PPM have reported an incidence of 0.7% to 1.8% after TAVR. The prognostic impact of PPM after TAVR has been variously reported and remains controversial [17, 23, 24]. In our study, when an iEOA of < 0.65 cm^2^/m^2^ was defined as severe PPM and 0.65–0.85 cm^2^/m^2^ was defined as moderate PPM, severe PPM occurred in 2 (1.0%) patients and moderate PPM in 32 (17.5%) patients. The incidence of PPM was significantly lower in the supra-annular implantation group, with no cases of severe PPM in this group. Several factors may be associated with PPM, including age, BSA, a small annulus, and BPD [23, 25]. Our multivariate analysis for avoiding PPM (iEOA > 0.85 cm^2^/m^2^) showed that supra-annular implantation was a stronger predictor than BMI and BPD. To avoid miscalculation of the EOA caused by the left ventricular outflow tract or annular diameter, we added a hemodynamic assessment based on the DVI using a > 0.5 for no PPM, and the results were similar to those of the iEOA. However, this threshold remains uncertain. A recent report of the PARTNER trial investigating normal THV function showed that the expected DVI for the SAPIEN 3 valve is > 0.43 [26]. Our multivariate analysis for an acceptable DVI with a cut-off of 0.43 similarly showed that supra-annular implantation was the independent factor. A small annulus is a predictor of hemodynamic valve dysfunction and PPM. For such patients, a supra-annular self-expandable valve is often used to provide a larger EOA. In fact, a recent randomized controlled trial involving patients with a small annulus (< 430 mm^2^) confirmed hemodynamic structural valve dysfunction in 32.8% of the BE valves but in only 3.5% of the self-expandable valves [8]. Therefore, supra-annular implantation might be particularly useful when these patients are treated with a BE valve.

Although supra-annular implantation of the SAPIEN 3 provides a low PPI rate and better hemodynamic function, it is associated with a risk of THV embolization, PVL, and coronary occlusion. However, there was no difference between the two groups in our study with regard to valve embolization and the need for a second valve within the first THV. Despite the performance of extremely high implantation with a mean implantation depth of 1.1 mm, mild PVL occurred in only two (1.6%) patients. We believe it is possible to implant the SAPIEN 3 in a supra-annular position, leading to better hemodynamic outcomes without an increase in the risk of valve embolization or PVL. It is important to note that the risk plane was significantly higher in the supra-annular BE-TAVR group with 63% of patents having a risk plane above STJ. When performing high THV implantation, the clinician must consider the difficulty of coronary re-access or the need for a TAV-in-TAV procedure following TAVR based on the anatomical assessment by pre-procedural CT.

## Study limitations

Important limitations of this study are the small sample size and the retrospective, single-center design. The THV frame diameter and depth were calculated by fluoroscopy; however, co-axiality between the annular plane and the THV plane was not always maintained. Although additional CT assessment might have been ideal, post-TAVR CT analysis could not be performed because data were lacking in the conventional implantation group. Additionally, few patients with a smaller annulus were included, possibly leading to PPM bias. PPM is more likely to occur in patients with a small annulus, and a self-expanding THV is often used for these patients. Further investigation is needed to determine the effect of the implantation depth on patients with a small annulus. In this study, a larger EOA and lower incidence of PPM were confirmed in supra-annular implantation, whereas peak velocity and mean PG showed no significant difference. Several factor, such as in-stent flow acceleration or a greater pressure recovery within the aorta, may affect the hemodynamic parameters of echocardiography. To validate the hemodynamic efficacy of supra-annular implantation, it is important to evaluate its prognostic value. In the present study, supra-annular implantation did not show a significant impact on one-year hospitalization and mortality. However, PPM may have a prognostic impact in long-term. Follow-up echocardiographic data and information on long-term clinical outcomes might be necessary to evaluate the prognostic impact of supra-annular implantation.

## Conclusions

In this single-center, retrospective analysis, supra-annular implantation of the SAPIEN 3 valve provided better hemodynamic performance compared with intra-annular implantation. Greater THV expansion contributes to this better hemodynamic function, and such expansion is more effective when the THV is deployed above the annular line. The complication rates of supra-annular implantation, including PVL and valve embolization, were noninferior to those of intra-annular implantation.

## Supplementary Information

Below is the link to the electronic supplementary material.

Supplementary file1 (DOCX 16 kb)

## Data Availability

The authors confirm that the data supporting the findings of this study are available within the article and its supplementary materials.

## References

[1] Mack MJ, Leon MB, Thourani VH, Makkar R, Kodali SK, Russo M, et al. Transcatheter aortic-valve replacement with a balloon-expandable valve in low-risk patients. N Engl J Med. 2019;380:1695–705. 30883058 10.1056/NEJMoa1814052

[2] Popma JJ, Deeb GM, Yakubov SJ, Mumtaz M, Gada H, O’Hair D, et al. Transcatheter aortic-valve replacement with a self-expanding valve in low-risk patients. N Engl J Med. 2019;380:1706–15. 30883053 10.1056/NEJMoa1816885

[3] Takeji Y, Taniguchi T, Morimoto T, Shirai S, Kitai T, Tabata H, et al. In-hospital outcomes after SAVR or TAVI in patients with severe aortic stenosis. Cardiovasc Interv Ther. 2024;39:65–73. 37349628 10.1007/s12928-023-00942-x PMC10764526

[4] Nijhoff F, Abawi M, Agostoni P, Ramjankhan FZ, Doevendans PA, Stella PR. Transcatheter aortic valve implantation with the new balloon-expandable Sapien 3 versus Sapien XT valve system: a propensity score-matched single-center comparison. Circ Cardiovasc Interv. 2015;8: e002408. 26033967 10.1161/CIRCINTERVENTIONS.115.002408

[5] Winter MP, Bartko P, Hofer F, Zbiral M, Burger A, Ghanim B, et al. Evolution of outcome and complications in TAVR: a meta-analysis of observational and randomized studies. Sci Rep. 2020;10:15568. 32968104 10.1038/s41598-020-72453-1 PMC7511292

[6] Okuno T, Khan F, Asami M, Praz F, Heg D, Winkel MG, et al. Prosthesis-patient mismatch following transcatheter aortic valve replacement with supra-annular and intra-annular prostheses. JACC Cardiovasc Interv. 2019;12:2173–82. 31564593 10.1016/j.jcin.2019.07.027

[7] Mauri V, Kim WK, Abumayyaleh M, Walther T, Moellmann H, Schaefer U, et al. Short-term outcome and hemodynamic performance of next-generation self-expanding versus balloon-expandable transcatheter aortic valves in patients with small aortic annulus: a multicenter propensity-matched comparison. Circ Cardiovasc Interv. 2017;10:e005013. 28951395 10.1161/CIRCINTERVENTIONS.117.005013

[8] Herrmann HC, Mehran R, Blackman DJ, Bailey S, Möllmann H, Abdel-Wahab M, et al. Self-expanding or balloon-expandable TAVR in patients with a small aortic annulus. New England J Med. 2024;21:1959–71. 10.1056/NEJMoa2312573 38587261

[9] Veulemans V, Maier O, Zeus T. Factors influencing implantation depth during transcatheter aortic valve replacement. Interv Cardiol. 2024;19(e01):2024. 10.15420/icr.2023.05 PMC10918527 38464494

[10] Mauri V, Reimann A, Stern D, Scherner M, Kuhn E, Rudolph V, et al. Predictors of permanent pacemaker implantation after transcatheter aortic valve replacement with the SAPIEN 3. JACC Cardiovasc Interv. 2016;9:2200–9. 27832845 10.1016/j.jcin.2016.08.034

[11] Ishizu K, Shirai S, Kawaguchi T, Taniguchi T, Hayashi M, Isotani A, et al. Effect of radiolucent line-guided balloon-expandable transcatheter aortic valve implantation on subsequent pacemaker rate. Am J Cardiol. 2022;165:72–80. 34895870 10.1016/j.amjcard.2021.11.010

[12] Sammour Y, Banerjee K, Kumar A, Lak H, Chawla S, Incognito C, et al. Systematic approach to high implantation of SAPIEN-3 valve achieves a lower rate of conduction abnormalities including pacemaker implantation. Circu Cardiovasc Interv. 2021;14:9407. 10.1161/CIRCINTERVENTIONS.120.009407 33430603

[13] Kim WK, Renker M, Doerr O, Hofmann S, Nef H, Choi YH, et al. Impact of implantation depth on outcomes of new-generation balloon-expandable transcatheter heart valves. Clin Res Cardiol : Off J German Cardiac Soc. 2021;110:1983–92. 10.1007/s00392-021-01932-w 34476559

[14] Ramanathan PK, Nazir S, Elzanaty AM, Nesheiwat Z, Mahmood M, Rachwal W, et al. Novel method for implantation of balloon expandable transcatheter aortic valve replacement to reduce pacemaker rate—line of lucency method. Struct Heart. 2020;4:427–32.

[15] Zoghbi WA, Jone PN, Chamsi-Pasha MA, Chen T, Collins KA, Desai MY, et al. Guidelines for the evaluation of prosthetic valve function with cardiovascular imaging: a report from the american society of echocardiography developed in collaboration with the society for cardiovascular magnetic resonance and the society of cardiovascular computed tomography. J Am Soc Echocardiogr. 2024;37:2–63. 38182282 10.1016/j.echo.2023.10.004

[16] Lancellotti P, Pibarot P, Chambers J, Edvardsen T, Delgado V, Dulgheru R, et al. Recommendations for the imaging assessment of prosthetic heart valves: a report from the european association of cardiovascular imaging endorsed by the chinese society of echocardiography, the inter-american society of echocardiography, and the brazilian department of cardiovascular imaging. Eur Heart J Cardiovasc Imaging. 2016;17:589–90. 27143783 10.1093/ehjci/jew025

[17] Hahn RT, Douglas PS, Jaber WA, Leipsic J, Kapadia S, Thourani VH, et al. Doppler velocity index outcomes following surgical or transcatheter aortic valve replacement in the PARTNER trials. JACC Cardiovasc Interv. 2021;14:1594–606. 34217631 10.1016/j.jcin.2021.04.007

[18] Généreux P, Piazza N, Alu MC, Nazif T, Hahn RT, Pibarot P, et al. Valve academic research consortium 3: updated endpoint definitions for aortic valve clinical research. J Am Coll Cardiol. 2021;77:2717–46. 33888385 10.1016/j.jacc.2021.02.038

[19] Ochiai T, Yamanaka F, Shishido K, Moriyama N, Komatsu I, Yokoyama H, et al. Impact of high implantation of transcatheter aortic valve on subsequent conduction disturbances and coronary access. JACC Cardiovasc Interv. 2023;16:1192–204. 37225290 10.1016/j.jcin.2023.03.021

[20] Simonato M, Azadani AN, Webb J, Leipsic J, Kornowski R, Vahanian A, et al. In vitro evaluation of implantation depth in valve-in-valve using different transcatheter heart valves. EuroIntervention. 2016;12:909–17. 27639744 10.4244/EIJV12I7A149

[21] Kawaguchi T, Yamaji K, Ishizu K, Morinaga T, Hayashi M, Isotani A, et al. Effect of postdilatation following balloon expandable transcatheter aortic valve implantation. Catheter Cardiovasc Interv. 2020;96:E630-e639. 31880388 10.1002/ccd.28676

[22] Nara Y, Watanabe Y, Kataoka A, Nakashima M, Hioki H, Kawashima H, et al. Balloon post-dilatation improves long-term valve performance after balloon-expandable valve implantation. Cardiovasc Revas Med. 2022;37:15–22. 10.1016/j.carrev.2021.06.008 34175251

[23] León Del Pino MDC, Ruíz Ortiz M, Delgado Ortega M, Sánchez Fernández J, Ferreiro Quero C, Durán Jiménez E, et al. Prosthesis-patient mismatch after transcatheter aortic valve replacement: prevalence and medium term prognostic impact. Int J Cardiovasc Imaging. 2019;35:827–36. 30661140 10.1007/s10554-018-01519-z

[24] Zorn GL 3rd, Little SH, Tadros P, Deeb GM, Gleason TG, Heiser J, et al. Prosthesis-patient mismatch in high-risk patients with severe aortic stenosis: a randomized trial of a self-expanding prosthesis. J Thorac Cardiovasc Surg. 2016;151(1014–1022):1023.e1011-1013. 10.1016/j.jtcvs.2015.10.070 26614412

[25] Miyasaka M, Tada N, Taguri M, Kato S, Enta Y, Otomo T, et al. Incidence, predictors, and clinical impact of prosthesis-patient mismatch following transcatheter aortic valve replacement in asian patients: the OCEAN-TAVI registry. JACC Cardiovasc Interv. 2018;11:771–80. 29673509 10.1016/j.jcin.2018.01.273

[26] Hahn RT, Leipsic J, Douglas PS, Jaber WA, Weissman NJ, Pibarot P, et al. Comprehensive echocardiographic assessment of normal transcatheter valve function. JACC Cardiovasc Imaging. 2019;12:25–34. 29909110 10.1016/j.jcmg.2018.04.010

