# Supplementary material for: Hemodynamic effect of supra-annular implantation of SAPIEN 3 balloon expandable valve
Source: Cardiovasc Interv Ther. 2024 Sep 6;40(1):133–43. doi: 10.1007/s12928-024-01040-2 (PMC11723897; doi:10.1007/s12928-024-01040-2)
Supplement: Supplementary file 1 — Supplementary file1 (DOCX 16 kb) [file 12928_2024_1040_MOESM1_ESM.docx]

***Supplemental table 1. Baseline characteristics and hemodynamic performance in patent with or without balloon post-dilatation***

|  | **BPD (-)**  **(N=48)** | **BPD (+)**  **(N=136)** | **p value** |
| --- | --- | --- | --- |
| ***Baseline characteristics*** | | | |
| Age, years | 86 (83-90) | 84 (81-88) | 0.02 |
| Male, n (%) | 15 (31.2.) | 55 (40.4) | 0.25 |
| BSA, m^2^ | 1.39 (1.29-1.51) | 1.45 (1.34-1.59) | 0.05 |
| LVEF, % | 65 (60-71) | 64 (56-70) | 0.79 |
| AVA, cm^2^ | 0.69 (0.60-0.79) | 0.74 (0.59-0.87) | 0.14 |
| BNP, pg/dl | 237.8 (138.6-414.7) | 221.6 (93.6-534.4) | 0.13 |
| Annulus area on CT, cm^2^ | 397.8 (350.8-467.3) | 410.9 (371.2-475.6) | 0.23 |
| Oversizing ratio, % | 6.9 (1.1-13.5) | 8.4 (4.1-13.6) | 0.17 |
| Valve Size, n (%)  20mm  23mm  26mm  29mm | 6 (12.5)  26 (54.1)  15 (31.2)  1 (2.0) | 3 (2.2)  73 (53.6)  51 (37.5)  9 (6.6) | 0.03 |
| ***Hemodynamic performance*** | | | |
| V peak, m/s | 2.3 (2.1-2.6) | 2.3 (2.1-2.6) | 0.53 |
| mean PG, mmHg | 11.5 (9-14) | 11 (9-14) | 0.58 |
| iEOA mean, cm^2^/m^2^ | 1.04 (0.85-1.20) | 1.07 (0.94-1.26) | 0.14 |
| DVI, mean | 0.51 (0.43-0.58) | 0.50 (0.42-0.59) | 0.88 |

Abbreviations: BSA, body surface area; LVEF, left ventricular ejection fraction; AVA, aortic valve area; BNP, brain natriuretic peptide; STS score, Society of Thoracic Surgery score; CT, computed tomography; PG, pressure gradients; EOA, effective orifice area; DVI, doppler velocity index.

**Supplemental table 2. *Predictor for favorable transcatheter heart valve function (indexed EOA>0.85): model 2***

|  | *Multivariate analysis* | |
| --- | --- | --- |
|  | OR (95% CI) | p-value |
| Age | 0.99 (0.93-1.06) | 0.86 |
| Male | 0.80 (0.36-1.77) | 0.58 |
| BSA (per 0.1m^2^ increase) | 1.15 (0.93-1.43) | 0.18 |
| Reduced EF (<50%) | 0.57 (0.22-1.44) | 0.23 |
| Pre-dilatation | 0.85 (0.34-2.08) | 0.72 |
| Post-dilatation | 0.42 (0.14-1.13) | 0.08 |
| THV Supra position | 4.65 (2.00-11.55) | <0.01 |
| Overfilling (vs nominal) | 1.02 (0.26-3.98) | 0.97 |
| Overfilling (vs underfilling) | 1.11 (0.28-4.46) | 0.87 |
| Nominal volume (vs underfilling) | 1.09 (0.51-2.37) | 0.81 |

Abbreviations: BSA, body surface area; EF, ejection fraction; THV, transcatheter heart valve.
